# Effect of Heat Stress on Dairy Cow Performance and on Expression of Protein Metabolism Genes in Mammary Cells

**DOI:** 10.3390/ani10112124

**Published:** 2020-11-16

**Authors:** Mirco Corazzin, Elena Saccà, Giovanna Lippe, Alberto Romanzin, Vinicius Foletto, Francesco Da Borso, Edi Piasentier

**Affiliations:** Department of Agricultural, Food, Environmental and Animal Sciences, University of Udine, 33100 Udine, Italy; mirco.corazzin@uniud.it (M.C.); giovanna.lippe@uniud.it (G.L.); alberto.romanzin@uniud.it (A.R.); vinicius.foletto@uniud.it (V.F.); Francesco.daborso@uniud.it (F.D.B.); edi.piasentier@uniud.it (E.P.)

**Keywords:** heat stress, mammary gland, somatic cell, mRNA, Western blot, protein synthesis

## Abstract

**Simple Summary:**

Environmental temperatures are increasing, and consequent global warming also has negative effects on dairy cattle farms, which may result in reduced production and poorer milk quality. The protein content of casein, in particular, is important in influencing the coagulation properties of milk and, therefore, the production and quality of cheese. The aim of this study was to assess the effect of heat stress on animal performance and on the expression of selected genes involved in milk protein metabolism. Eight dairy cows were kept under thermoneutral conditions for 8 days. The same animals were then maintained under mild heat stress conditions for an additional 8 days. The results of this study revealed that mild heat stress reduced the feed intake and performance of dairy cows in terms of milk and protein yield, but not the expression of the target genes involved in milk protein metabolism, such as those coding for caseins.

**Abstract:**

The aim of this study was to assess the effect of heat stress on dairy cow performance and on the expression of selected genes involved in milk protein metabolism. Eight Italian Holstein Friesian cows were kept under thermoneutral conditions (temperature–humidity index (THI) < 72, CON) for 8 days and under mild heat stress conditions (72 < THI < 78, HS) for an additional 8 days. The rectal temperature, feed intake, and milk yield were recorded during the last 3 days of the CON and HS periods. During the same time period, milk samples were collected to assess the composition and expression of selected genes involved in milk protein metabolism. Gene expression analyses were performed on somatic cells from milk, which are representative of mammary tissue. In terms of dairy cow performance, HS resulted in lower milk and protein yields and feed intake but higher rectal temperature than for CON (*p* < 0.05). Under HS, there were greater abundances of HSPA1A (*p* < 0.05) and BCL2 (*p* < 0.05), compared to CON, but similar levels of CSN2 (*p* > 0.05), CSN3 (*p* > 0.05), HSPA8 (*p* > 0.05), and STAT5B (*p* > 0.05) mRNA. Mild heat stress reduced the performance of dairy cows without affecting the expression of genes coding for caseins.

## 1. Introduction

Global warming and climate change are among the biggest issues facing the world, and their economic impact on dairy farming is a relevant issue. The reduction in milk yield related to heat stress has led to estimated losses of 5.4% of the monthly income of farmers during summer [1]. This impact is expected to significantly increase in the future, with the annual average land temperature in Europe expected to increase by 1.0–5.5 °C by the end of the century [2].

The temperature–humidity index (THI) [3] is widely used to predict heat stress events in cattle, and a value of 72 points was initially considered to be the threshold at which heat stress begins [4]. This threshold was later reduced to 68 points for dairy cows that produce more than 35 kg/d [5]. The content of true protein in milk is important in terms of determining the primary income of farmers and also the dairy industry due to its influence on milk coagulation properties and, hence, cheese production and quality [6]. Heat stress can have a detrimental effect not only on dairy cow performance but also on the milk content of true protein and particularly caseins [7]. These changes could be the result of the indirect effect of reduced dry matter intake and of the direct effect (tissue hyperthermia) on mammary synthesis [8]. There are many studies available which investigate the effect of heat stress on the performance of dairy cows [9,10]. West et al. [11] explained that every additional 1 °C in air temperature above the thermal neutral zone causes a 0.85 kg reduction in feed intake, and that heat stress can cause a considerable reduction in milk production. Fewer studies have characterized the effects of heat stress on milk protein composition or protein fractions, and the results are often contradictory [7,8]. The effects of heat stress on the expression of genes involved in the synthesis of milk proteins are far from being well understood, and few in vivo studies are available [12,13]. When measured in vitro, heat stress increased the expression of heat shock proteins (Hsp) and of genes that code for milk proteins [14,15].

In the past, many studies on gene expression in bovine mammary glands were performed on mammary tissue collected at slaughter or through mammary biopsies that were invasive, did not ensure animal welfare, required surgical procedures, or did not allow for repetitive sampling without injuring the mammary gland. With the aim of avoiding these problems, one study demonstrated that somatic cells from milk are an effective source of mammary transcripts [16]. To our best knowledge, only one study that used milk somatic cells for assessing the effect of heat stress on gene expression in mammary cells is available [17], and this study was performed on goats.

It has previously been hypothesized that heat stress could also have a detrimental effect on milk composition by reducing the expression of target genes in mammary glands. Therefore, the aim of this study was to assess the effect of heat stress on milk production and composition in dairy cows, and on the expression of selected genes involved in milk protein metabolism in milk somatic cells.

## 2. Materials and Methods

The study was conducted in accordance with EU Directive 2010/63/EU and Italian legislation (DL n. 26, 4 March 2014), and adhered to the rules of the University of Udine. No invasive procedures were applied, and the adopted procedures were routine. The ethical committee of the University of Udine approved the trial (prot. no. 4/2017).

### 2.1. Animals, Treatments and Sampling

**Figure 1:**
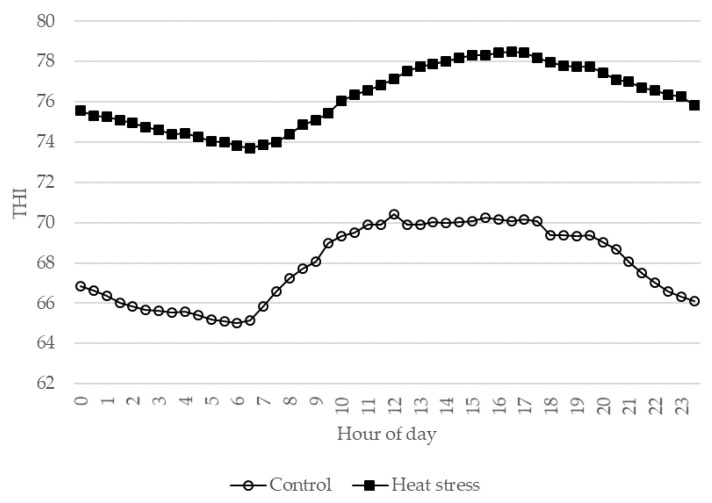
Average diurnal patterns of temperature–humidity index (THI) for control and heat stress conditions.

Eight multiparous Italian Holstein Friesian dairy cows in the last stage of lactation and belonging to one commercial farm were considered and transferred to the experiment farm of the University of Udine (Azienda Agraria Universitaria Antonio Servadei) where the trial took place. At the beginning of the trial, the animals had 271 ± 3.7 (mean ± SE) days of milk production, a mean milk production of 14.7 ± 0.84 kg, and a mean milk somatic cell count (SCC) of 131,625 ± 18,643 cells/mL. All animals had an SCC lower than 200,000 cells/mL, which indicated that the mammary gland was free from infection or physiological stress [18,19]. The animals were housed in a tie-stall barn equipped with a fan cooling system and 4 mini data loggers (FT-102; Econorma SAS, Treviso, Italy) that monitored the environmental temperature and relative humidity every second, and recorded averages every thirty minutes. The THI was calculated [3] on the basis of these data considering the hourly average value measured by the probes. The dimensions of the barn were 25 × 10 m with an average height of 4 m. After an adaptation period of 3 weeks, the animals were kept under thermoneutral conditions (CON; THI < 72) for 8 days with a fan used to help maintain these conditions. The same animals were then kept under environmental barn conditions that corresponded to mild heat stress conditions (HS) (THI ranging from 72 to 78 [4]) for an additional 8 days. The average diurnal patterns of the THI during CON and HS conditions are reported in Figure 1. The cows were in good health and had regular veterinary inspections during the entire adaptation and experiment period. The animals were offered sorghum silage, 6 kg of dry matter (DM)—concentrate, 9.2 kg of DM—and hay ad libitum. The utilized concentrate consisted of maize (426 g/kg), soybean meal (215 g/kg), wheat bran (157 g/kg), sunflower meal (81 g/kg), wheat middlings (78 g/kg), and minerals and vitamins (44 g/kg), and this was fed to the cows twice daily during milking. The diet was formulated in order to meet the nutritional requirements of dairy cows in accordance with the Institut National de la Recherche Agronomique (INRA) standard [20]. Samples of hay, silage, and concentrate were collected every two days; dried at 65 °C in a forced draft oven for 48 h; and analyzed for dry matter (DM), ash, ether extract (EE), crude protein (CP) [21], and neutral detergent fiber (NDF) [22]. The energetic value of the feeds, expressed as net energy for lactation (NE_L_), was estimated following the INRA standard [20]. The ingredients and chemical composition of the feed offered to the dairy cows are reported in Table 1. The daily individual dry-matter intake (DMI) of hay, silage, and concentrate was determined by weighing the amounts of offered and refused feed. During the entire experiment period, the rectal temperature (RT) of the animals was measured three times a day (at 6:00, 14:00, and 22:00 h) with a thermometer (Digital thermometer, GIMA, Milan, Italy) inserted to a depth of 3 cm for at least 3 min. Individual daily milk yield was recorded, and individual milk samples were collected from all four quarters of every cow during morning milking for analyses of composition and gene expression. The milk intended for RNA analysis was filtered with sterile gauze, collected in sterile containers kept on ice, and immediately processed following sample collection. The individual values for the rectal temperature, milk variables (yield, composition, and protein fractions), feed intake, and gene expression obtained during the last 3 days of the CON and HS periods were averaged for each individual, and the result values were used in data analysis. The milk yield was also expressed as fat-corrected milk (FCM) [23].

### 2.2. Milk Composition and Protein Fractions

Samples obtained from 50 mL of milk were analyzed for fat, protein, and lactose according to ISO 9622:2013 using a MilkoScan FT6000 (FOSS Electric, Hillerød, Denmark) [24], and for somatic cell count (SCC), using a Foss-o-Matic (FOSS Electric, Hillerød, Denmark) according to ISO 1366-2:2008 [25].

Samples obtained from 50 mL of milk were analyzed for milk protein fractions by sodium dodecyl sulfate polyacrylamide gel electrophoresis (SDS-PAGE) according to Laemmli [26] with modifications. Samples of defatted milk were prepared by adding an equal volume of double-concentrated (2×) sample buffer (0.125 mol/L Tris-HCl, pH 6.8, 4% SDS, 20% glycerol, 10% 2-mercaptoethanol, and 0.2% bromophenol blue), and heated at 98 °C for 5 min. Electrophoresis was performed in a Mighty Small II Mini Vertical Electrophoresis System (Hoefer, Holliston, MA, USA). A stacking gel was prepared with 4% (*v/v*) acrylamide-N,N′-methylenebisacrylamide (2% Bis Solution, Bio-Rad) at a 32:1 (*w/w*) ratio, solubilized in 0.125 mol/L Tris-HCl buffer, pH 6.8, with 0.1% (*w/v*) SDS. The running gel contained 13% acrylamide-N,N′-methylenebisacrylamide at a 36:1 (*w/w*) ratio, solubilized in 0.375 mol/L Tris-HCl, pH 8.8, with 0.1% (*w/v*) SDS; 0.1% (*w/v*) ammonium persulfate and 0.025% (*v/v*) N,N,N′,N′-tetramethylethylenediamine (TEMED) were added in both gel solutions to initiate polymerization. Electrophoresis was performed at a constant voltage (30 V) until the samples completely left the stacking gel, and then at a constant current of 15 mA (and 250 V) until the tracking dye reached the bottom of the running gel. After electrophoresis, the gels were placed under oscillation in a fixing solution (50% methanol and 10% acetic acid) for 1 h and subsequently placed in a staining solution (10% acetic acid and 0.25% (*w/v*) Coomassie Blue G-250) overnight. The gels were then destained in a 10% acetic acid solution. Protein fractions were identified on the basis of their electrophoretic behavior [27]. The band molecular weights were estimated by comparison with a molecular weight (MW) protein standard in the range of 10–250 kDa (Precision Plus Protein Dual Color Standards; Bio-Rad). The protein content of the bands from milk were estimated through comparison with bovine serum albumin (BSA) solutions of known concentrations ranging 0.25–2.5 µg/µL; the standard solutions was electrophoresed in a separate lane. Gel image acquisition was performed using the G:Box instrument and Gene Sys software, version 1.5.7.0 (Syngene, Cambridge, UK). The molecular weight estimation and protein quantification of the separated bands were performed with the GeneTools software, version 4.3.9.0 (Syngene).

### 2.3. Milk Somatic Cell Isolation

Two liters of milk per animal was collected in RNase-free tubes and processed for RNA isolation and Western blot analyses.

For the assessment of somatic cell (SC) gene expression, total RNA was first extracted using the method of Tudisco et al. [28] with modifications. Milk samples were divided into 500 mL tubes (four tubes per animal) and SCs were pelleted by centrifugation at 1500× *g* for 15 min at 4 °C. The cell pellets were gently washed 3–5 times with 5–10 mL of phosphate-buffered saline (PBS) buffer (pH 7.2) with 0.5 mmol/L ethylenediaminetetraacetic acid (EDTA), to eliminate casein and fat globules, and 0.1% diethylpyrocarbonate (DEPC). After washing, the pellets were gently resuspended in 5–10 mL of PBS–EDTA–DEPC solution, transferred into 50 mL tubes (pellets from two 500 mL tubes were combined into one 50 mL tube), and made up to 50 mL volume using PBS–EDTA–DEPC solution. The tubes were centrifuged at 1000× *g* for 10 min at 4 °C, and the supernatant was discarded. The cell pellets were resuspended with 100–200 µL PBS–EDTA–DEPC solution and were finally transferred to 2 mL tubes. A portion of the suspended cells was sent for RNA analysis, while the other was stored at −80 °C for protein immunodetection analysis.

### 2.4. RNA Analysis

RNA extraction was performed using TRI Reagent (Sigma-Aldrich, St. Louis, MI, USA), and deoxyribonuclease (DNase) treatment was carried out using a Precision DNase kit (PrimerDesign, Southampton, UK) according to the manufacturer’s protocols. The concentration and purity of the extracted RNA were assessed using a NanoDrop One spectrophotometer (Thermo Scientific, Waltham, MA, USA). RNA integrity and the absence of genomic DNA were assessed by agarose gel electrophoresis through comparison with MW standards to highlight the presence of only distinct and clear 18S and 28S rRNA bands. Complementary DNA (cDNA) was prepared using the iScript cDNA Synthesis kit (Bio-Rad, Hercules, CA, USA) according to the manufacturer’s protocol. Each 20 μL reaction contained 4 μL reverse transcription reaction mix, 1 μL iScript reverse transcriptase, the appropriate volume of RNA solution required for 50 ng/μL final RNA concentration, and the corresponding volume of nuclease-free water to make up the final volume. The mixture was incubated for 5 min at 25 °C, 60 min at 42 °C, and 5 min at 85 °C before being cooled on ice. Qualitative polymerase chain reaction (PCR) was carried out on a sample pool to validate primer pair specificity for all target genes (β-casein, CSN2; k-casein, CSN3; heat shock protein family A (Hsp70) member 1A, HSPA1A; heat shock protein family A (Hsp70) member 8, HSPA8; B-cell lymphoma 2, BCL2; mammalian target of rapamycin, MTOR; signal transducer and activator of transcription 5B, STAT5B; and reference genes (Table 2)). PCR was performed using the Bio-Rad CFX96 system (Bio-Rad) on a reaction volume of 20 μL containing 0.3 μL of each forward and reverse primer (0.3 μmol/L), 10 μL SsoAdvanced Universal SYBR Green Supermix (Bio-Rad), 8.4 μL sterile water, and 1 μL cDNA. The amplification conditions were one cycle of 3 min at 95 °C; 40 PCR cycles of 15 s at 95 °C, 30 s at 60 °C, and 30 s at 72 °C; and 1 min at 95 °C, followed by a melt curve of 55–95 °C with 0.5 °C increments every 5 s. The occurrence of nonspecific amplification and primer–dimer formation was excluded by checking the melt curves. The amplicon length was verified by agarose gel electrophoresis, comparing with MW standards (Table 2). Quantitative PCR (qPCR) was performed using the same instrument, reagents, and volumes as those for qualitative PCR. Each sample was analyzed in triplicate, and relative gene expression was calculated according to the efficiency-corrected 2^−ΔΔCt^ method [29,30]. β-Actin (ACTB), glyceraldehyde-3-phosphate dehydrogenase (GAPDH), and ribosomal protein large P0 (RPLP0) were initially considered as reference genes. ACTB, being the most stable gene, was used for the normalization of the qPCR data. The data are presented as fold-change ratios using the CON group as a reference. The primer efficiency was calculated using the standard curve obtained by the amplification of the serial dilution of the pooled cDNA [29] (Table 2).

### 2.5. Immunodetection of Bcl2 and Hsp70 in Somatic Cells

The expression levels of two proteins of interest, Bcl2 and Hsp70, were assessed by SDS-PAGE followed by Western blotting. SC samples were prepared by adding 0.25 vol of 5× sample buffer (0.5 mol/L Tris-HCl, pH 6.8, 10% SDS, 50% glycerol, 25% 2-mercaptoethanol, and 0.2% bromophenol blue) and heating at 98 °C for 5 min. SDS-PAGE was performed as described above. The proteins were then transferred to a nitrocellulose membrane (Hybond ECL; Amersham Pharmacia Biotech, Little Chalfont, UK) using blotting apparatus (Novex X Cell II Blot Module—X Cell Sure Lock Electrophoresis Cell; Life Technologies, Carlsbad, CA, USA). The transfer was performed at 30 V (and 150 mA) for 45 min. After staining with Ponceau S solution (0.1% (*w/v*) Ponceau S, 5% acetic acid) to verify transfer efficiency, the nitrocellulose sheets were saturated with 5% (*w/v*) nonfat dry milk in washing buffer (1× PBS, 0.1% Tween 20) for 2 h at room temperature under oscillation. After blocking, the nitrocellulose sheets were washed with washing buffer, cut at 37 kDa in the case of Bcl2 detection and at 50 kDa in the case of Hsp70-Hspa1a detection, and then incubated with the primary specific antibodies at 4 °C overnight under oscillation. For Bcl2 immunodetection, the upper membrane part was probed with antibodies against Actin (anti-Actin, Cat. N. A2066; Sigma-Aldrich; 42 kDa) at a concentration of 1:2000, while the lower part was probed with antibodies against Bcl2 (anti-Bcl2, N. SAB4500003; Sigma-Aldrich; 25 kDa), diluted 1:500. For Hsp70 immunodetection, the upper membrane part was probed with antibodies against Hsp70 (Anti-Hspa1a, N. SAB2107600; Sigma-Aldrich; 70 kDa) diluted 1:500, while the lower part was probed with antibodies against Actin diluted 1:2000. All blots were then rinsed three times with washing buffer and incubated for 2 h at room temperature with the peroxidase-coupled secondary antibody anti-rabbit-IgG (N. SAB3700934; Sigma Aldrich) diluted 1:5000. The membranes were then washed three times in washing buffer and developed with ECL Star (Enhanced Chemiluminescent Substrate; EuroClone, Pero, Mi, Italy), according to the manufacturer’s protocol. The acquisition of blot images was performed with the G:Box instrument and Syngene software (Syngene, Cambridge, UK). The quantification of the proteins was performed with the GeneTools software (Syngene), measuring the band intensity and normalizing it to that of Actin.

### 2.6. Statistical Analysis

Statistical analysis was performed using R software v. 3.4.0 [38]. The normality of data distribution was tested using the Shapiro–Wilk test. Differences in the means for all the variables between CON and HS were assessed using a paired-sample t-test. In cases where the assumptions of this test were not satisfied, the Wilcoxon signed-rank test was used. Values are reported as mean ± SE; a *p* value less than 0.05 was considered to indicate significant differences, while a *p* value less than 0.10 was considered as a tendency towards statistical significance.

## 3. Results and Discussion

Heat stress increased RT (*p* < 0.01; Table 3) by 0.5 °C on average. An increase in RT therefore indicates the effectiveness of heat stress [39]. Heat stress is the condition in which dairy cows are not able to completely dissipate the heat load that is derived from their metabolism and the environment [11]; consequently, rectal temperature can increase. In agreement with the results of the present study, Bouraoui et al. [40] observed an increase of 0.5 °C (from 38.4 to 38.9 °C) in the average daily RT of low-producing Holstein Friesian dairy cows when the THI increased from 68 to 78 points. Ting et al. [41] reported rectal temperature values of 39.1 and 38.3 °C in animals maintained for 4 days at a THI greater than 76 and in animals not in conditions of heat stress, respectively. Moreover, RT is significantly correlated with THI, and even variations of less than 1 °C can negatively affect the milk yield and DMI of dairy cattle [42,43]. Karimi et al. [39] explained that the effect of heat stress on DMI is mediated by RT increases. Indeed, HS resulted in lower total DMI than for CON (*p* < 0.05; Table 4), by about 14%. However, this significant reduction involved the forage fraction, observed for both silage (23%, *p* < 0.05; Table 4) and hay (30%, *p* < 0.05; Table 4) but not concentrate (*p* > 0.05). Garner et al. [44] explained that a reduction in ingestion is an adaptive mechanism for high temperatures that allows the animal to reduce metabolic heat production derived from rumen fermentation. However, the production of endogenous heat due to rumen fermentation is influenced by feed characteristics. Generally, a diet rich in fiber can increase the production of endogenous heat compared to a diet rich in concentrates. Indeed, Coppock and West [45] found that by increasing the hay in diets from 50% to 100%, the conversion efficiency of the metabolizable energy in milk was reduced from 65% to 34%. In agreement with our results, Cowley et al. [8], in considering dairy cows after 7 days of heat stress (a THI of approximately 78), observed a 14% reduction in ingestion. Bouraoui et al. [40] observed a 10% reduction in DMI in heat-stressed dairy cows. Under HS, lower milk and FCM yields were observed compared to those under CON (*p* < 0.05; Table 3), by about 18% and 13%, respectively. Polsky et al. [46] explained that the reduction in milk production as a result of high THI values can be due to both a reduction in DMI and an increase in the maintenance needs of the animal, which can reach +25%. Many authors found a reduction in milk production in heat-stressed dairy cows. Pragna et al. [47] reviewed that heat stress could lead to a reduction in milk production of up to 30%–40%, and the magnitude of this reduction was positively related to the intensity of the heat stress and milk yield [48]. Ting et al. [41] observed a 19% reduction in milk yield in high-producing dairy cows that had undergone 4 days of heat stress.

Considering milk composition, heat stress tended to increase the content of protein (*p* < 0.10) and fat (*p* < 0.10), but not the content of lactose (*p* > 0.10; Table 3). At the same time, HS reduced the daily protein yield (*p* < 0.05) without affecting the daily fat yield (*p* > 0.05; Table 3). The review by Tao et al. [49] concluded that the effect of heat stress on milk fat and protein content is largely inconsistent. In the present study, the tendency toward higher percentage of protein and fat in the milk of heat-stressed cows could be due to the reduction in milk yield and subsequent concentration of protein and fat in addition to possibly greater non-protein nitrogen contents in the milk produced from cows under HS rather than CON. Similar to the present study, Cowley et al. [8] observed that dairy cows under heat stress had reduced daily protein yields. The SCC tended to be higher under HS than CON (*p* < 0.10; Table 3). Bernabucci et al. [7] also showed a tendency for SCC increase in milk collected in summer in comparison to milk collected in the winter or spring season, whereas Bouraoui et al. [40] observed that the SCC increased significantly as a result of heat stress. Total milk casein (*p* > 0.05) as well as β-casein, κ-casein, and whey protein (*p* > 0.05) were not affected by heat stress. Conversely, αs-casein tended to be lower in milk from cows under HS than CON (*p* < 0.10; Table 3). The average values of casein and whey protein concentrations were similar to those reported by Bernabucci et al. [7] during summer, while the milk casein reported by Cowley et al. [8] was slightly higher at 27.5 g/L. The average values of αs-casein and κ-casein were higher and lower, respectively, than those reported by Cowley et al. [8] by 50.1% and 11.0%, respectively. Barlowska et al. [50] reported that the relationship between α_S_-casein and κ-casein can vary. The proportion of κ-casein observed in the present study is comparable to that reported by Islam et al. [51] (7%), who explained that the proportion of k-casein to whole casein is highly variable in relation to many factors, such as genetic polymorphism, the stage of lactation, and diet. Conflicting results about the effect of heat stress on milk protein fractions are reported in the literature. Bernabucci et al. [7] reported reduced milk casein and α_S_-casein concentration in the summer season, though κ-casein was increased without any effect on β-casein when expressed as a percentage of total casein. Cowley et al. [8] reported that heat stress reduced milk casein and αs2-casein concentrations, with no effect on κ-casein, but milk whey protein concentrations increased. More recently, Ma et al. [52] failed to detect differences in casein fractions (α_S_-, β-, and κ-casein) in dairy cows exposed to heat stress (82.4 for the THI).

The effect of heat stress on the mRNA abundance of genes involved in milk protein metabolism in milk somatic cells is reported in Table 5. Under HS, there was significantly higher mRNA expression of the HSPA1A (*p* < 0.05) and BCL2 (*p* < 0.05) genes and a tendency for higher expression of MTOR (*p* < 0.10) gene than under CON. An effect of heat stress was not found on the mRNA expression of the CSN2, CSN3, HSPA8, and STAT5B genes (*p* > 0.05). The significant effects of heat stress on BCL2 and HSPA1A expression were also supported by the results of Western blot analysis (Table 6). Indeed, the protein expression of BCL2 (*p* < 0.05) and HSPA1A (*p* < 0.05) genes was higher under HS than CON. The HSPA8 gene codes for Hsc70 (known as heat shock cognate 71 kDa protein), which is 86% identical to Hsp70 encoded by HSPA1A [53]. The expression of HSPA8 was not affected by heat stress; indeed, Hsc70 is constitutively expressed, having a key role in the cell in protein folding and unfolding, and it can be considered as a housekeeping gene [53,54]. As expected, Hsp70 was highly expressed during heat stress. Its role is to protect the cell and bind proteins to prevent their aggregation, and it has an antiapoptotic function [55]. In agreement with the results of the present study, Salama et al. [56] and Hu et al. [14] observed an increase in HSPA1A gene expression and the mRNA abundance of Hsp70 in mammary cells incubated at 42 °C in comparison with cells incubated at 36/38 °C. Considering other genes involved in apoptosis, heat stress increased the expression of BCL2, which is an antiapoptotic gene. In their vitro study, Hu et al. [57] observed that under heat stress, cells increased the expression of antiapoptotic factors to reduce heat damage. Under HS, there was tendency for higher MTOR mRNA expression than under CON. Kaufman et al. [58] observed that heat stress reduced MTOR signaling pathway activity in mammary cells in vitro. Conversely, Salama et al. [56] failed to find an effect of heat stress on the mRNA abundance of MTOR in bovine mammary cells in vitro. On the other hand, Chou et al. [59] observed that MTOR has a key role in the synthesis of Hsp; in particular, a reduction in MTOR is accompanied by a reduction in the synthesis of Hsp70; in this way, cells become more sensitive to heat shock. The induction of the gene expression of STAT5 is crucial for the expression of the gene coding for β-casein, CSN2 [33,60]. For this reason, an effect of heat stress on the expression of the CSN2 gene was not found. Moreover, the expression of the gene coding for κ-casein, CSN3, was not affected by heat stress, in agreement with the results for κ-casein in milk.

## 4. Conclusions

In the present study, dairy cows in the last stage of lactation that were under mild heat stress clearly had reduced daily milk, feed intake, and protein yield. However, little difference was observed in the casein and whey proteins of milk. The expression of selected genes involved in milk protein metabolism in mammary cells was characterized in milk somatic cells, which allow for repetitive sampling without injuring the mammary gland. From this point of view, mRNA and Western blot analyses showed that mammary cells under mild heat stress had increased expression of the antiapoptotic genes HSPA1A and BCL2, probably involved in the protection from heat damage, and the mRNA abundance of genes coding for caseins was not affected. The results of the present study expand our knowledge of the animal’s responses to heat stress and thus lay the foundations for improving or introducing breeding techniques that can improve dairy cow welfare and production.

## Figures and Tables

**Table 1:** Ingredients and chemical composition of feed offered to dairy cows.

Item	Hay	Sorghum Silage	Concentrate
Ingredient offered (kg DM/d)	Ad libitum	6	9.2
Chemical composition			
DM (%)	90.4	27.5	87.6
Ash (% DM)	7.4	6.10	8.5
EE (% DM)	1.6	2.4	4.8
CP (% DM)	7.6	6.9	20.5
NDF (% DM)	71.2	58.8	23.8
NE_L_ (MJ/kg DM)	4.7	5.8	7.6

DM: dry matter; EE: ether extract; CP: crude protein; NDF: neutral detergent fiber; NE_L_: net energy for lactation.

**Table 2:** Primer sequences, amplification products, and amplification efficiency for the target genes and reference genes.

Gene	Primers Sequence, 5′ to 3′	Amplicon Length (bp)	Accession Number (NCBI; GenBank)	Efficiency (%)	R^2^
CSN2 ^1^	F: CCCTAACAGCCTCCCACA R: AGCCATAGCCTCCTTCAC	112	KC993858.1	101.1	0.996
CSN3 ^2^	F: TGCAATGATGAAGAGTTTTTTCCTAG R: GATTGGGATATATTTGGCTATTTTGT	150	NM_174294.2	97.1	0.999
HSPA1A ^3^	F: AACATGAAGAGCGCCGTGGAGG R: GTTACACACCTGCTCCAGCTCC	171	NM_203322.3	101.0	0.988
HSPA8 ^4^	F: CGAATCATCAATGAGCCAACTG R: TGCCACCCCCTAAATCAAAG	100	NM_174345.4	102.0	0.999
BCL2 ^5^	F: TGTGGATGACCGAGTACCTGAA R: GACAGCCAGGAGAAATCAAACAG	124	NM_001166486.1	100.6	0.997
MTOR ^6^	F: CGTTCCTCTCAACATGGACACA R: AGCTTCTCCGCGTCTTTACAA	102	XM_002694043.5	96.3	0.988
STAT5B ^7^	F: GCCAACAATGGTACTTCTCCG R: TGTGTGACCAGTCGCAGCTC	101	NM_174617.4	98.5	0.997
ACTB ^8^	F: CTCTTCCAGCCTTCCTTCCT R: GGGCAGTGATCTCTTTCTGC	177	NM_173979.3	100.4	0.999
GAPDH ^9^	F: TCATCCCTGCTTCTACTGGC R: CCTGCTTCACCACCTTCTTG	176	NM_001034034	100.7	0.998
RPLP0 ^10^	F: CAACCCCGAAGTGCTTGACAT R: AGGCAGATGGATCAGCCA	226	NM_001012682.1	100.2	0.999

CSN2: β-casein; CSN3: κ-casein; HSPA1A: heat shock protein 70 isoform A1A; HSP8: heat shock protein 70 isoform A8; BCL2: B-cell lymphoma 2; MTOR: mammalian target of rapamycin; STAT5B: signal transducer and activator of transcription 5B; ACTB: β-Actin; GAPDH: glyceraldehyde-3-phosphate dehydrogenase; RPL0: ribosomal protein large P0; F: forward; R: reverse. References: ^1^ Hu et al. [14]; ^2,5^ Boutinaud et al. [31]; ^3^ Kapila et al. [15]; ^4^ Li et al. [32]; ^6^ Yang et al. [33]; ^7^ Li et al. [34]; ^8^ Duckett et al. [35]; ^9^ Bernabucci et al. [36]; ^10^ Wang et al. [37].

**Table 3:** Effect of heat stress on rectal temperature, milk yield, milk composition, and protein fractions of dairy cows (*n* = 8).

Item	Treatment Group		SE	*p*-Value
	CON	HS		
Rectal temperature (°C)	38.6	39.1	0.116	0.002
Milk yield (kg/d)	14.6	12.0	0.75	0.009
FCM (kg/d)	14.4	12.6	0.607	0.020
Milk composition				
Protein (%)	3.27	3.37	0.043	0.057
Fat (%)	3.83	4.33	0.262	0.097
Lactose (%)	4.44	4.50	0.099	0.529
SCC × 1000 (cells/mL)	128	197	35.4	0.091
Milk total casein (%)	2.20	2.14	0.610	0.416
αs-casein (% total casein)	60.77	59.53	0.572	0.066
β-casein (% total casein)	32.92	34.17	0.669	0.105
κ-casein (% total casein)	6.30	6.30	0.448	0.998
Milk whey protein (%)	0.97	0.98	0.021	0.734
Protein yield (g/d)	481	400	23.9	0.012
Fat yield (g/d)	568	518	27.0	0.111

CON: thermoneutral conditions (temperature–humidity index < 72); HS: heat stress conditions (temperature–humidity index > 72); SE: standard error of the difference; FCM: fat-corrected milk; SCC: somatic cell count.

**Table 4:** Effect of heat stress on daily intake of dairy cows (*n* = 8).

Item	Treatment Group		SE	*p*-Value
	CON	HS		
Concentrate (kg DM)	8.61	8.30	0.238	0.641
Forage (kg DM)	7.66	5.67	0.613	0.014
Sorghum silage (kg DM)	4.39	3.37	0.270	0.007
Hay (kg DM)	3.27	2.29	0.397	0.044
Total (kg DM)	16.26	13.97	0.771	0.021

CON: thermoneutral conditions (temperature–humidity index < 72); HS: dairy heat stress conditions (temperature–humidity index > 72); SE: standard error of the difference; DM: dry matter.

**Table 5:** Fold change ratio for relative mRNA expression of genes involved in milk protein metabolism (*n* = 8) in dairy cows ^1^.

Gene	Treatment Group		SE	*p*-Value
	CON	HS		
CSN2	1	2.35	0.495	0.215
CSN3	1	4.68	0.904	0.164
HSPA8	1	1.11	0.278	0.461
HSPA1A	1	1.28	0.362	0.020
BCL2	1	1.66	0.403	0.016
MTOR	1	3.00	0.542	0.059
STAT5B	1	2.43	0.507	0.129

CON: thermoneutral conditions (temperature–humidity index < 72); HS: heat stress conditions (temperature–humidity index > 72); SE: standard error of the mean; CSN2: β-casein; CSN3: κ-casein; HSPA1A: heat shock protein 70 isoform A1A; HSP8: heat shock protein 70 isoform A8; BCL2: B-cell lymphoma 2; MTOR: mammalian target of rapamycin; STAT5B: signal transducer and activator of transcription 5B. ^1^ β-Actin was used for data normalization.

**Table 6:** Fold change ratio, relative to Actin, of protein expression of BCL2 and HSPA1A genes in dairy cows (*n* = 8).

Gene	Treatment Group		SE	*p*-Value
	CON	HS		
BCL2	0.100	0.133	0.0050	0.007
HSPA1A	0.170	0.304	0.0482	0.049

CON: thermoneutral conditions (temperature–humidity index < 72); HS: heat stress conditions (temperature–humidity index > 72); SE: standard error of the difference; BCL2: B-cell lymphoma 2; HSPA1A: heat shock protein family A (Hsp70) member 1A.
